# Toward feeling, understanding, and caring: The development of empathy in young autistic children

**DOI:** 10.1177/13623613221117955

**Published:** 2022-08-23

**Authors:** Boya Li, Els Blijd-Hoogewys, Lex Stockmann, Ilaria Vergari, Carolien Rieffe

**Affiliations:** 1 Leiden University, The Netherlands; 2 INTER-PSY, The Netherlands; 3 Twente University, The Netherlands; 4 University College London, UK

**Keywords:** attention, autism spectrum disorders, early childhood, emotion acknowledgment, emotion contagion, empathy development, longitudinal, prosocial action

## Abstract

Autistic people are often portrayed as lacking empathy. Yet they are not indifferent to others’ feelings. To advance our understanding of the early development of empathy in autistic children, this longitudinal study followed the development of four empathy abilities: emotion contagion, attention to others, emotion acknowledgment, and prosocial actions, in 1- to 6-year-old autistic children (*N* = 61; M_age_ = 55.49 months), in comparison with non-autistic peers (*N* = 145; M_age_ = 52.16 months). Once a year, for 4 consecutive years, children’s empathic reactions were evaluated by experimenters who acted out emotional episodes to elicit empathy in children, and by parents who filled out empathy questionnaires. We confirmed autistic children’s difficulty attending to others, acknowledging others’ emotions, and initiating prosocial actions. However, according to parents, they did not differ from non-autistic children in emotion contagion with others’ negative emotions. Notably, autistic children showed a greater increase in prosocial actions over time than their non-autistic peers. We discussed how to interpret these findings in light of the “double empathy problem,” and stressed the importance of removing the stereotypical view of autism. Furthermore, this study was among the first to show that autistic children have the potential to learn and to improve empathy skills.

**Lay abstract:**

Empathy is a highly valued human capacity. Yet, autistic people are often portrayed as lacking in empathy. Recent research, which views empathy as a complex construct emerging from multiple interrelated emotional and cognitive processes, argues that, although many autistic people do have difficulty understanding others’ emotions, and this may hinder them from responding to others in a prosocial manner, they are not indifferent to other people’s feelings. Hoping to contribute to a better understanding of the unique challenges that autistic children face in their empathy development, we followed the development of four empathy abilities: emotion contagion, attention to others, emotion acknowledgment, and prosocial actions, in 1- to 6-year-old autistic children, in comparison with non-autistic children. Once a year, for 4 consecutive years, children’s empathy abilities were evaluated by experimenters who acted out emotional episodes to provoke empathy in children, and by parents who filled out empathy questionnaires. We found that autistic children experienced indeed more difficulty attending to others, acknowledging others’ emotions, and initiating prosocial actions toward others. However, according to parents, they did not differ from their non-autistic peers in feeling along with others’ negative emotions. This indicates that it might not be the case that autistic children did not want to act empathetically toward others. Rather, they might not know how to do so. Notably, despite these difficulties, when looking at children’s developmental trajectories, autistic children showed similar improvements over time as non-autistic children. This provides evidence that autistic children have the potential to learn and to improve their empathy skills.

While empathy is a highly valued human capacity, a conventional view of autism often portrays autistic people as lacking in empathy. Recent research, which frames empathy as a multicomponent construct, has advanced the notion that, although many autistic people do have difficulty understanding others’ emotions, and this difficulty may hinder them from responding to others in a prosocial manner, autistic people are not indifferent to other people’s feelings (Fletcher-Watson & Bird, 2020). This multicomponent approach to empathy may help mitigate the negative stereotypical view of autism (Santiesteban et al., 2021), and facilitates an understanding of the unique challenges that autistic people face in their development of empathy (Bons et al., 2013). Information in this regard obtained from autistic children at young ages can be of particular importance (Zwaigenbaum et al., 2015).

Although there is lack of a universal definition of empathy (Hall & Schwartz, 2019), four interrelated emotional and cognitive processes are often discussed in empathy research (Bird & Viding, 2014; Decety & Moriguchi, 2007; Overgaauw et al., 2017; Rieffe et al., 2010; Tousignant et al., 2017). Observing the emotional state, especially the negative emotions, of another person, can induce an echoing emotional state in oneself (“emotion contagion”) (Decety & Meyer, 2008; Rieffe et al., 2021). Emotion contagion is observed already in early infancy: babies who were only a few months old became upset when witnessing another person in distress (Roth-Hanania et al., 2011). Although the emotional distress is induced by contagiously catching another person’s distress, since young children have difficulty regulating emotional arousals, they themselves can become overwhelmed (Davidov et al., 2013). Therefore, a crucial step in the empathy process is to keep one’s own emotions under control and to switch the focus of attention to another (“attention to others”) (Bird & Viding, 2014). For young children, not immersed in one’s own distress but paying close attention to another is a starting point for understanding others’ emotions (“emotion acknowledgment”) (Tsou et al., 2021). Accordingly, longitudinal studies examining empathy development in early childhood found that, while emotion contagion increased only slightly or remained stable, attention to others and emotion acknowledgment kept developing with age (Davidov et al., 2013; Tousignant et al., 2017). Furthermore, when one feels another’s emotions, pays attention to them and understands them, the person is motivated to and empowered to respond prosocially (“prosocial actions”) (Eisenberg et al., 2010). Prosocial actions such as helping and comforting in response to another’s distress have been found to grow in quantity and quality during early childhood (Flook et al., 2019; Zahn-Waxler et al., 1992).

While empathy seems to develop naturally and effortlessly in most typically developing children, it is more challenging for autistic children. First, diminished attention to the emotional display of another person has been reported in empathy research on autistic children aged 1–7 years (Campbell et al., 2015; Corona et al., 1998; Dawson et al., 2004; Hutman et al., 2010). These studies used behavioral tasks, where children’s empathic reactions to the emotional display of an adult were observed and evaluated. Autistic children were found to pay less attention to the adult researcher’s emotional display than did non-autistic children.

Reduced attention to social stimuli such as people, faces, and body movements is observed in autistic individuals across the life span and across contexts (for a review, see Chita-Tegmark, 2016). Some researchers have ascribed this to a diminished social motivation, positing that social interactions are less rewarding for autistic individuals, and that they therefore orient less often and less spontaneously toward other people (Chevallier et al., 2012). However, this account contradicts the testimonies of autistic individuals, who have claimed to long for social interactions (Jaswal & Akhtar, 2019). Furthermore, neurophysiological research found that, instead of having hypo-reactivity, autistic individuals experienced hyper-reactivity when exposed to social stimuli such as direct gazes and emotional expressions (e.g. Dalton et al., 2005; Monk et al., 2010). It has been proposed that autistic people avert their attention as a regulating strategy to avoid being overwhelmed by what they experience as intense social input (Markram & Markram, 2010; Tanaka & Sung, 2016).

Not attending to another person in an empathy-provoking situation, either voluntarily or spontaneously, may amplify the existing difficulties that autistic people experience in their understanding of the cognitive mind (Theory of Mind (ToM)) and the emotional mind of others (emotion acknowledgment) (Wieckowski & White, 2020). Note, however, that problems in ToM and emotion acknowledgment may be bidirectional. As stated by the “double empathy problem,” when it comes to understanding the autistic mind, non-autistic people can also be blind and lost (Milton, 2012). In line with this, the misattunement theory advocates that the social difficulties and communication breakdowns that autistic people encounter may result from a mismatch of communication styles and a mutual misunderstanding between autistic and non-autistic people (Bolis et al., 2017; Davis & Crompton, 2021).

This misattunement account may explain, at least in part, the inconsistent findings on emotion contagion in autistic people. The abovementioned empathy research on autistic children, which used observational tasks, all reported a lower level of emotion contagion among autistic children than in non-autistic peers. However, studies using parent reports (Deschamps et al., 2014; Hudry & Slaughter, 2009) and self-reports (Dziobek et al., 2008; Mul et al., 2018; Pouw et al., 2013; Santiesteban et al., 2021) found no group differences in emotion contagion. In addition, as mentioned earlier, comparable or higher levels of physiological arousals related to empathy were found in autistic people, when their attention to the social stimuli was maintained (Dijkhuis et al., 2019; Fan et al., 2014; Hadjikhani et al., 2017; Trimmer et al., 2017). This raises the following question: Could autistic children’s emotional sharing with others be underestimated by non-autistic researchers in the observational studies? Emerging evidence has shown that non-autistic people indeed struggle in interpreting the emotion expressions of autistic people (Brewer et al., 2016; Sheppard et al., 2016). They tend to view autistic people as not approachable and eccentric during initial encounters, even without knowing their diagnosis (Sasson et al., 2017).

However, even if emotion contagion is intact in autistic children, given their reduced attention to others and difficulties in acknowledging others’ emotions, it is not surprising that fewer prosocial actions are often observed in autistic children as compared with non-autistic children (e.g. Hudry & Slaughter, 2009; Russell et al., 2013). Two studies did not find group differences, which evaluated children’s prosocial reactions to the emotional display of parents (McDonald & Messinger, 2012) and of a virtual player in computerized tasks (Deschamps et al., 2014). Possibly, compared with the social demand of reacting to an adult stranger as in other empathy studies, it is less stressful for autistic children to react to their parents or to a virtual player.

As discussed so far, empirical evidence gathered from cross-sectional studies shows that in an empathy-provoking situation, autistic children may in fact not lack the ability to feel for others. However, they may still experience difficulty attending to, understanding, and reacting prosocially toward others. Albeit informative, the cross-sectional nature of these studies precludes an evaluation of the developmental course of empathy in autistic children.

To date, only a few longitudinal studies checked the development of some empathy components in young autistic children. First, regarding emotion contagion, a stable trend in a short term of 6 months (McDonald & Messinger, 2012; Zantinge, 2018) and an increasing trend in a long term of 3 years (Hutman et al., 2010) were found in autistic toddlers. Besides, attention to others and prosocial actions were observed to increase in autistic children (Hutman et al., 2010; McDonald & Messinger, 2012; Russell et al., 2013; Zantinge, 2018). Regarding emotion acknowledgment, although the ability improves with age in autistic children (e.g. Rosen & Lerner, 2016; Steel et al., 2003), the magnitude of improvement might be less than in non-autistic children. Past research found that while perspective-taking abilities such as ToM and emotion acknowledgment improved over time in both autistic and non-autistic children, the improvement in autistic children was with a slower rate than in non-autistic children (e.g. Peterson et al., 2005). In addition, the difference between autistic and non-autistic peers widens from early childhood to adolescence and adulthood (Harms et al., 2010; Lozier et al., 2014).

## Present study

This four-wave longitudinal study aimed to investigate the development of four components of empathy, that is, emotion contagion, attention to others, emotion acknowledgment, and prosocial actions, in autistic children aged 1–6 years, in comparison with non-autistic peers.

Prior research showed that autistic children were viewed as more empathic by parents than by experimenters in observational tasks (Deschamps et al., 2014; Hudry & Slaughter, 2009). While parents may have better insight than experimenters into children’s idiosyncratic and nuanced emotional reactions, they also tend to overestimate their child’s empathic abilities (Neary, 2022). Yet, the assessment by experimenters provides a snapshot of autistic children’s reactions to others in real-life situations and can inform us how autistic children’s reactions are perceived by non-autistic people other than parents. Considering that any single measure provides only a partial assessment of the underlying construct and can limit the explanatory power of the results (Kienbaum, 2014), we assessed empathy using both parent questionnaires and observational tasks. Parents evaluated the four empathy abilities in their children through questionnaires, and experimenters acted out emotional episodes and observed children’s reactions in observational tasks. Since it was difficult to incorporate a test of emotion acknowledgment in real time, only emotion contagion, attention to others, and prosocial actions were measured in the observational tasks.

Based on the literature presented above, we expected autistic children to pay less attention to others, to show less emotion acknowledgment, and to display fewer prosocial actions than their non-autistic peers. We also expected these group differences to be maintained over time. Regarding emotion contagion, we expected experimenters to evaluate autistic children as showing less emotion contagion, and parents to report equivalent levels of emotion contagion for autistic and non-autistic children alike.

Regarding the developmental trajectories, for non-autistic children, we expected their emotion contagion to show either a small increase or to remain stable over time, while we expected their attention to another, emotion acknowledgment, and prosocial actions to increase with age. Due to limited evidence from longitudinal data on autistic children, our hypotheses regarding their empathy development were exploratory. We expected autistic children to show similar developmental trajectories of emotion contagion, attention to others, and prosocial actions to their non-autistic peers. Regarding emotion acknowledgment, we expected it to increase in autistic children, but we expected an increase that would be of a smaller magnitude than in non-autistic children.

## Methods

### Participants and procedure

This study was part of a larger-scaled longitudinal research in the Netherlands on the socioemotional development of preschool children with limited access to the social world, including children with hearing loss (Tsou et al., 2021), with developmental language disorder (Rieffe & Wiefferink, 2017), and with autism (Li et al., 2021). The total sample of the larger-scaled research included 73 Dutch children with autism (65 boys) and 418 Dutch children (226 boys) without autism. Autistic children met the following inclusion criteria: having an autism diagnosis according to the *Diagnostic and Statistical Manual of Mental Disorders* (4th ed.; DSM-IV; American Psychiatric Association, 2000), backed up by the *Autism Diagnostic Interview-Revised* (Lord et al., 1994), and set by a qualified child psychologist or psychiatrist at Time 1; a confirmation by parents 3 years later that the child retained the diagnosis; the child had IQ scores above 70; and no additional *Diagnostic and Statistical Manual of Mental Disorders* (4th ed., text rev.; DSM-IV-TR) diagnoses. Inclusion criteria for non-autistic children were IQ scores above 70 and no DSM-IV-TR diagnoses.

Autistic children were recruited via a specialized institution that provided diagnosis and intervention for autism, Center for Autism, Leiden, The Netherlands. Non-autistic children were recruited from daycare centers and mainstream schools in the same region. Since the IQ profiles of autistic children were either retrieved from school or collected by the institution, various intelligence tests were used, including Snijders-Oomen Nonverbal Intelligence Test (SON-R; Snijders et al., 1989), Wechsler Intelligence Scale for Children (Wechsler, 1974), Wechsler Preschool and Primary Scale of Intelligence (Wechsler, 1990), and Wechsler Nonverbal Scale of Ability (WNV; Wechsler & Naglieri, 2006). Non-autistic children were tested with the SON-R.

The Ethics Committee of Leiden University and Center for Autism granted permission for the larger-scaled research project (P08.140/SH/sh). All parents provided written informed consent. Children and their parents participated in the research once a year for 4 consecutive years (mean duration between Time 1 and 2 = 13.15 months, SD = 3.31; between Time 2 and 3 = 12.13 months, SD = 1.58; between Time 3 and 4 = 12.37, SD = 1.06). Children were visited either at school or at the specialized institution (for the autistic participants only), where they finished a series of tasks under the guidance of a psychologist who had received training for administering these tasks and for coding children’s behaviors. Parents filled out questionnaires to report on their children’s development. The Social Responsive Scale (SRS; Constantino & Gruber, 2005) were filled out at Time 1, Time 3, and Time 4, where parents reported on the degree of their children’s autistic features. The SRS consists of 65 items with responses on a 4-point scale, where higher scores indicate more accentuated autistic traits. First, raw total scores were calculated. Then, the raw scores were converted to *T* scores according to the Dutch SRS manual (Roeyers et al., 2011).

Due to time constraints, not all children were administered the full battery of tasks. Participants of the larger research project were included in this study if we had data for them for the examined variables at least one time point (see Supplementary Tables 1 and 2 for available data at each time point). The final sample included 61 autistic children and 145 non-autistic children.

Table 1 shows the descriptive characteristics of the two groups. Autistic and non-autistic children did not differ in age (1.21 < *t*s < 1.74, *p*s > 0.05) or gender distribution (χ^2^(1) = 0.81, *p* = .367). Autistic children had on average lower IQs than non-autistic children (*t*(83.22) = 2.21, *p* = .03). Autistic children scored higher on the SRS scale than non-autistic children at Time 1(*t*(37.87) = 11.21, *p* < 0.001), Time 3 (*t*(63.63) = 9.09, *p* < 0.001), and Time 4 (*t*(48.19) = 9.51, *p* < 0.001). Mothers of autistic children had on average lower education levels than mothers of non-autistic children (*t*(95.04) = 3.03, *p* = 0.003), whereas the education levels of fathers did not differ (*t*(89.95) = 1, *p* = 0.32). Families of autistic children had lower income than families of non-autistic children (*t*(130) = 3.64, *p* < 0.001).

**Table 1.** Demographic characteristics of participants: M (SDs) of background variables.

		Total participants at Time 1			
		*N* = 206			
		Autistic	*N*	Non-autistic	*N*
			61		145
Age in months	Time 1	55.49 (12.64)	61	52.16 (12.50)	145
	Time 2	66.90 (13.19)	49	66.57 (13.48)	51
	Time 3	80.67 (11.80).	46	77.54 (13.29)	48
	Time 4	93.38 (11.83)	40	88.95 (13.37).	41
Male		88.5%	54	92.4%	134
IQ*		99.08 (16.46)	50	105.14 (11.03)	59
SRS *T* score	Time 1**	75.13 (11.42)	40	46.92 (6.32)	13
	Time 3**	72.92 (20.40)	51	45.21 (7.33)	47
	Time 4**	78.23 (15.77)	31	47.06 (9.16)	31
Education mother^a^,*		3.82 (1.13)	52	4.43 (0.87)	48
Education father^a^		3.79 (1.28)	53	4.03 (0.96)	39
Net annual income^b^,**		2.96 (1.11)	43	3.74 (1.19)	89

SRS: Social Responsive Scale.

a Parental education level: 1 = no/primary education; 2 = lower general secondary education; 3 = middle general secondary education; 4 = higher general secondary education; 5 = college/university.

b Net household income: 1 = less than €15,000; 2 = €15,000–€30,000; 3 = €30,000–€45,000; 4 = €45,000–€60,000; 5 = more than €60,000.

* *p* < 0.05; **p < 0.001.

There was no community involvement in this reported study.

### Materials

Two parent questionnaires were used to evaluate the four empathy abilities. The Empathy Questionnaire (Rieffe et al., 2010) asks parents to evaluate the extent to which their children showed emotion contagion when others display negative emotions (6 items, e.g. “When another child cries, my child gets upset too”), attention to others (7 items, e.g. “When an adult gets angry with another child, my child watches attentively”), and prosocial actions (6 items, e.g. “When another child starts to cry, my child tries to comfort him/her”), over the past 2 months on a 3-point scale: 0 = not at all applicable; 1 = a little or sometimes applicable; 2 = clearly or often applicable.

The Emotion Expression Questionnaire (Li et al., 2020) asks parents to evaluate their children’s emotion expression and emotion acknowledgment. For the current study, we used the scale “Emotion acknowledgment” (6 items), where parents reported the extent to which their children recognized and understood happiness, anger, fear, sadness, and joy in their parents (e.g. “Does your child understand when you are happy?”) on a 5-point scale (from “1 = (almost) never applicable” to “5 = (almost) always applicable”).

Three Empathy Observational Tasks (EMT; Ketelaar et al., 2013) were administered to evaluate children’s empathic responses to the emotional display by the experimenter: at each time point, the experimenter acted out three different emotional episodes, where he or she pretended to be happy (e.g. clicking a pen while laughing aloud), angry (e.g. being mad at a pen which did not write), or in pain/distress (e.g. hurting a finger when closing a folder). Following each acting-out, children’s reactions were observed and rated using a 3-point scale (0 = not at all applicable; 1 = a little or sometimes applicable; 2 = clearly or often applicable). The coding schemes consisted of three scales: (1) emotion contagion (6 items; e.g. “The child shows similar emotions as the experimenter”), (2) attention to others (6 items; e.g. “The child stops playing and looks at the experimenter”), and (3) prosocial actions (4 items, not for the happy-emotion episodes; e.g. “The child tries to help”). The EMT and their coding schemes were designed based on the classical empathy task developed by Zahn-Waxler and colleagues (1992) for measuring empathic responses in toddlers and preschoolers.

With parents’ agreement, the administration of the EMTs was videotaped. The psychologists who administered the tasks rated children’s reactions during the experiment and reviewed their ratings through video recordings afterwards. All the participating psychologists had received intensive training on administering and coding the behaviors. They had achieved a high interrater reliability during practice before they went to work independently. In addition, one author took a random selection of 10% of participants (6 autistic children and 14 non-autistic children) and rated their behaviors from video recordings. The interrater agreements were good (Time 1: .80 < *k* < 1.00; Time 2: .84 < *k* < 1.00; Time 3: .81 < *k* < 1.00; Time 4: .81 < *k* < 1.00). A caveat should be mentioned. The raters were aware of the diagnosis of the autistic participants. It was difficult to mask the diagnosis because most autistic children did the tasks at the specialized institution where they were recruited. In addition, atypical behaviors of autistic children were easily noticeable.

The means, standard deviations, and reliabilities of all measurements are reported in Supplementary Tables 1 and 2. The questionnaires and EMTs showed satisfactory to good reliabilities across time (autistic group: 0.83 < *ω_t_* < 0.96; non-autistic group: 0.73 < *ω_t_* < 0.95).

### Statistical analyses

R (version 3.3.3; R Core Team, 2019) was used to check measurement reliabilities (with the package “psych”; Revelle, 2020) and to make figures (with the package “ggplot2”; Wickham, 2009). IBM SPSS Statistics for Macintosh (version 26.0; Armonk, NY, USA, IBM Corp.) were used to conduct linear mixed model (LMM) analyses for examining the developmental trajectories of empathy. LMM can account for the dependency within the longitudinal data (Hox et al., 2010) and is robust in handing randomly missing data (Twisk et al., 2013). The current data had missing values at every time point. Little’s MCAR (Missing Completely At Random) tests indicated that the missing patterns could have occurred completely at random (4407.23 < χ^2^s < 15,244.44, *p*s > 0.05).

We followed a formal model-fitting procedure, that is, fitting increasingly more complex models to the data, step-by-step. Simpler models with a better model fit were selected over a more complex model. To evaluate model fit, for nested models, likelihood ratio chi-square tests were conducted to select the preferred model, which showed significant less deviance, that is, lower values of −2 Log Likelihood (−2LL). For non-nested models, the preferred model showed lower Akaike information criterion (AIC) and Bayesian information criterion (BIC) values.

To examine the developmental trajectories of empathy, we started with a null model with only a fixed and random intercept. Then, age (centered around 21 months, the youngest age of all participants) was added to the model. We examined two models of change: linear and quadratic. Next, group (0 = non-autistic, 1 = autistic) was added to examine whether the levels of empathy differed between the two groups across time. Fourth, we added the interaction of age and group to the model to examine whether the two groups differed in developmental trajectories.

## Results

### Developmental trajectories of parent-evaluated empathy

**Figure 1.**
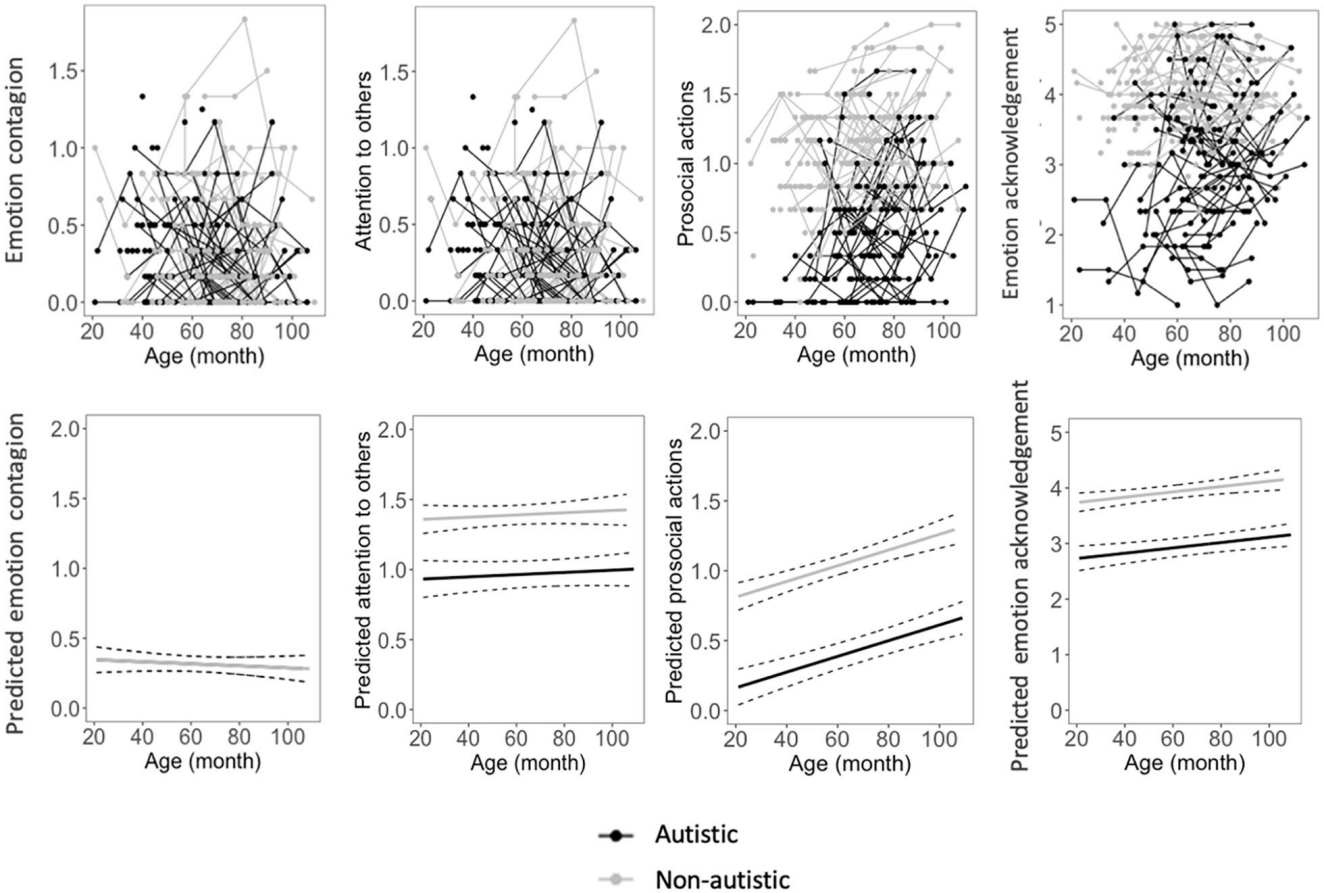
Developmental trajectories of parent-reported empathy. Top, from left to right: graphic representations of the levels of parent-reported emotion contagion, attention to others, prosocial actions, and emotion acknowledgment at four time points. The points were connected in lines, each line representing one participant. Participants who had data at one time point are presented by points. Bottom, from left to right: regression lines depicting predicted levels of parent-reported emotion contagion, attention to others, prosocial actions, and emotion acknowledgment with 95% CIs based on the best age models.

Estimates of the best age models for parent-reported empathy are reported in Table 2 (see also Supplementary Table 3). Developmental trajectories are depicted in Figure 1. For the development of parent-evaluated emotion contagion with others’ negative emotions, the best-fitting model was with the fixed effect of linear age (*t*(323.04) = −0.001, *p* = 0.348), indicating that emotion contagion with negative emotions did not change over time. Adding group did not contribute to a better model fit, indicating that parents reported equivalent levels of emotion contagion for the two groups.

**Table 2.** Fixed and random effects of the best age models for parent-reported empathy.

	Emotion contagion			Attention to others		
Fixed effects	Estimates (SE)	CI [low, high]	*p* value	Estimates (SE)	CI [low, high]	*p* value
Intercept	0.34 (0.04)	[0.27, 0.43]	<0.001	1.35 (0.05)	[1.25, 1.45]	<0.001
Age	−0.0008 (0.001)	[−0.002, 0.0008]	0.348	0.001 (0.001)	[−0.001, 0.003]	0.914
Group	–	–	–	−0.42 (0.06)	[−0.54, −0.30]	<0.001
Random effects	Estimates (SE)	CI [low, high]	Wald’s *Z*	Estimates (SE)	CI [low, high]	Wald’s *Z*
Residual	0.05 (0.01)	[0.04, 0.06]	10.05	0.06 (0.01)	[0.05, 0.07]	10.69
Intercept	0.06 (0.01)	[0.05, 0.08]	6.57	0.10 (0.01)	[0.08, 0.13]	7.10
	Prosocial actions			Emotion acknowledgment		
Fixed effects	Estimates (SE)	CI [low, high]	*p* value	Estimates (SE)	CI [low, high]	*p* value
Intercept	0.80 (0.05)	[0.71, 0.90]	<0.001	3.74 (.09)	[3.57, 3.91]	<0.001
Age	0.006 (0.001)	[0.004, 0.008]	<0.001	0.0035 (0.001)	[0.001, 0.008]	0.002
Group	−0.64 (0.06)	[−0.75, −0.53]	<0.001	−1.01 (0.10)	[−1.21, −0.80]	<0.001
Random effects	Estimates (SE)	CI [low, high]	Wald’s *Z*	Estimates (SE)	CI [low, high]	Wald’s *Z*
Residual	0.06 (0.01)	[0.05, 0.07]	10.49	0.16 (0.02)	[0.14, 0.19]	10.81
Intercept	0.10 (0.01)	[0.07, 0.13]	6.79	0.33 (0.04)	[0.25, 0.42]	7.58

SE: standard error; CI: confidence interval.

The best-fitting model for parent-reported attention to another person was with fixed effects of linear age (*t*(346.26) = 0.91, *p* = 0.361) and group (*t*(173.30) = −7.13, *p* < 0.001). These results indicate that attention did not change over time, and it was lower in autistic children overall.

For parent-reported prosocial actions, the best-fitting model was with fixed effects of linear age (*t*(345.83) = 6.22, *p* < 0.001) and group (*t*(164.15) = −11.25, *p* < 0.001), indicating that prosocial actions increased with age in all children. Yet, autistic children overall displayed fewer prosocial actions than non-autistic children.

For parent-reported emotion acknowledgment, the best-fitting model was with fixed effects of linear age (*t*(338.71) = 3.06, *p* = 0.002) and group (*t*(181.96) = −1.01, *p* < 0.001), indicating that while emotion acknowledgment increased with age in all children, overall, autistic children were evaluated as having lower emotion acknowledgment.

### Developmental trajectories of experimenter-evaluated empathy

**Figure 2.**
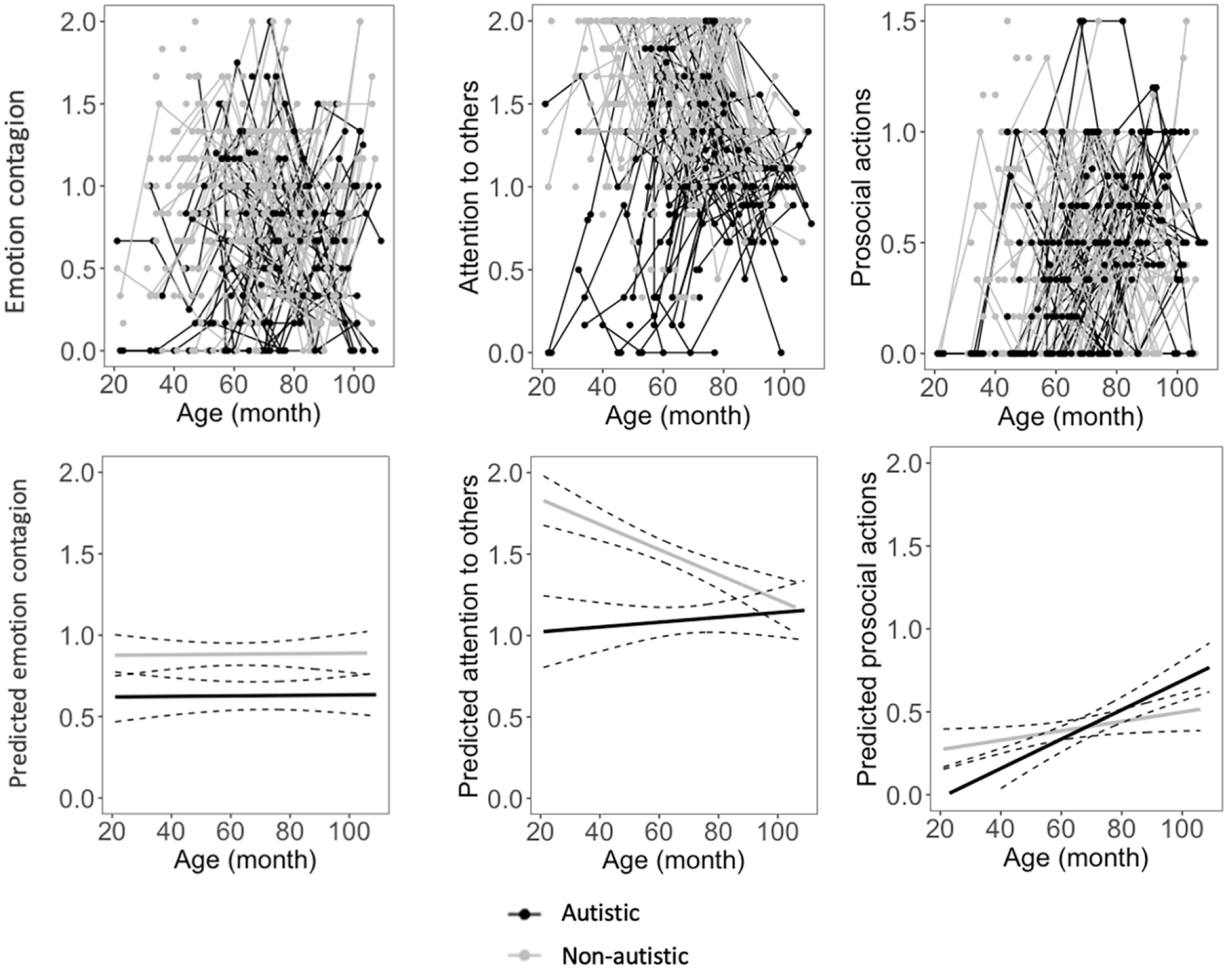
Developmental trajectories of empathy observed in tasks. Top, from left to right: graphic representations of the levels of observed emotion contagion, attention to others, and prosocial actions at four time points. The points were connected in lines, each line representing one participant. Participants who had data at one time point are presented by points. Bottom, from left to right: regression lines depicting predicted levels of observed emotion contagion, attention to others, and prosocial actions with 95% CIs based on the best age models.

Estimates of the best age models for experimenter-evaluated empathy are reported in Table 3 (see also Supplementary Table 3). Developmental trajectories are depicted in Figure 2. The best-fitting model for experimenter-evaluated emotion contagion was with the fixed effects of linear age (*t*(470.18) = 1.04, *p* = 0.301) and group (*t*(146.88) = −4.26, *p* < 0.001). This indicated that emotion contagion did not change over time, and that the experimenters rated lower levels of emotion contagion in autistic children than in non-autistic children.

**Table 3.** Fixed and random effects of the best age models for experimenter-reported empathy.

Emotion contagion				Attention to others			Prosocial actions		
Fixed effects	Estimates (SE)	CI [low, high]	*p* value	Estimates (SE)	CI [low, high]	*p* value	Estimates (SE)	CI [low, high]	*p* value
Intercept	0.81 (0.06)	[0.69, 0.92]	<0.001	1.79 (0.07)	[1.65, 1.92]	<0.001	0.26 (0.06)	[0.16, 0.37]	<0.001
Age	0.001 (0.001)	[−0.001, 0.004]	0.301	−0.008 (0.001)	[−0.01, −0.005]	<0.001	0.003 (0.001)	[0.001, 0.005]	0.012
Group	−0.23 (0.06)	[−0.34, −0.13]	<0.001	−0.77 (0.11)	[−1.01, −0.53]	<0.001	−0.24 (0.09)	[−0.42, −0.05]	0.013
Age * group	–	–		.009 (.002)	[0.004, 0.013]	<0.001	0.005 (0.002)	[0.001, 0.008]	0.010
Random effects	Estimates (SE)	CI [low, high]	Wald’s *Z*	Estimates (SE)	CI [low, high]	Wald’s *Z*	Estimates (SE)	CI [low, high]	Wald’s Z
Residual	0.17 (0.01)	[0.15, 0.20]	11.94	0.17 (0.02)	[0.14, 0.20]	15.48	0.11 (0.01)	[0.09, 0.13]	12.08
Intercept	0.05 (0.01)	[0.03, 0.09]	3.57	0.04 (0.02)	[0.02, 0.09]	2.74	0.02 (0.01)	[0.01, 0.05]	3.00

SE: standard error; CI: confidence interval.

The best-fitting model for experimenter-evaluated attention was with fixed effects of linear age (*t*(427.98) = −5.05, *p* < 0.001), group (*t*(445.52) = −6.36, *p* < 0.001), and the interaction between age and group (*t*(466.82) = 3.73, *p* < 0.001). Autistic children paid overall less attention than non-autistic children to the emotional display of the experimenter. Besides, attention decreased with age in non-autistic children (*b* = −0.006, *t*(284) = −4.55, *p* < 0.001), whereas it did not change in autistic children (*b* = 0.0006, *t*(194.82) = 0.28, *p* = 0.780).

The best-fitting model for experimenter-evaluated prosocial actions was with fixed effects of linear age (*t*(430.41) = 2.54, *p* = 0.012), group (*t*(448.01) = −2.51, *p* = 0.013), and the interaction between age and group (*t*(461.29) = 2.59, *p* = 0.010). Although autistic children showed overall fewer prosocial actions than non-autistic children, prosocial actions increased in autistic chil-dren over time with a greater magnitude (*b* = 0.008, *t*(191.38) = 5.88, *p* < 0.001) than in non-autistic children (*b* = 0.003, *t*(252.12) = 2.43, *p* = 0.016).

## Discussion

This four-wave longitudinal study was among the first to simultaneously address four key components of empathy and their development in young autistic children, using different informants. Consistent with the literature, we found a discrepancy between parent-evaluated and experimenter-evaluated emotion contagion in autistic children. That is, while parents reported equal levels of emotion contagion in autistic and non-autistic children, experimenters reported less emotion contagion in autistic children. In addition, lower levels of attention to others, emotion acknowledgment, and prosocial actions were found in autistic children. Regarding developmental trajectories, our findings aligned with the few existing longitudinal studies that showed that emotion contagion remained stable, whereas emotion acknowledgment and prosocial actions increased with age in autistic children. However, no age effect was found in attention to others. Furthermore, the developmental trajectories of attention to others and prosocial actions differed between autistic and non-autistic children.

First, our findings confirmed the literature on autistic children’s difficulties in attending to others, acknowledging others’ emotions, and responding prosocially toward others in empathy-provoking situations. Not understanding the emotions of another while being exposed to that person’s emotional display can be stressful for autistic children. To cope with this stress, they may switch their attention away, that is, “out of sight, out of mind” (Markram & Markram, 2010; Tanaka & Sung, 2016). Although avoiding emotional stimuli may be relieving at the moment, in the long run it is detrimental to the autistic child’s emotional development: a vicious cycle can occur, where struggles in emotion acknowledgment propel autistic children to avoid attending to others’ emotions; this hinders them from learning about emotions, which in turn leads to future and further avoidance of the emotional stimuli (Dawson et al., 2005). Furthermore, if an autistic child does not pay attention to a given situation and does not understand what is needed of them, how can we expect the child to react prosocially?

However, this is only one side of the story. The ratings of both the parent questionnaires and the observational tasks described empathic reactions that are typical of non-autistic children. They might not capture the autistic ways for communicating empathy. For example, autistic children were rated by both parents and experimenters as showing fewer prosocial actions as compared with non-autistic children. For both informants, prosocial actions were defined as comforting and helping behaviors, such as giving a hug or trying to cheer up another person. These behaviors are most observed in non-autistic people, especially in females. However, autistic people may express their empathy differently. For example, instead of focusing on the emotions, autistic people may focus on the problems and express empathy by searching for solutions (Rieffe et al., 2021). Some autistic people show their care and support by sitting quietly with another and listening patiently to another (Crompton et al., 2020).

In a similar vein, a possible explanation for the experimenter-rated lower level of emotion contagion in autistic children could be that autistic children’s vocal and facial emotion expressions differed from those of non-autistic children and thus were rated by experimenters as inconsistent or incorrect in social occasions (Capriola-Hall et al., 2019; Faso et al., 2015). In addition, as mentioned before, it was not possible to conceal the diagnosis of participants during the test situation. We cannot exclude the possibility that experimenters, even if unconsciously, might hold preempted expectations toward the empathic reactions of autistic children. Compared with experimenters, parents might be more familiar with their autistic child’s emotional reactions due to their daily and close interactions with the child.

The discrepancy between parent- and experimenter-evaluated emotion contagion may also result from the difference in settings for evaluation. In the observational tasks, the social demands of interacting with an adult stranger could be taxing for autistic children. Facing an adult stranger could provoke anxiety in these children. This in turn could disrupt their empathy process (Corbett et al., 2014; O’Connor et al., 2019). On the contrary, parents’ observations were based on their daily interactions with their child and the interactions of their child with other children. These situations were more relaxing and could invite more emotional responses from autistic children. It is also possible that autistic children were emotionally more involved with their parents and with peer acquaintances than with adult strangers, and thus their emotions resonated more with these familiar agents (Pierce & Redcay, 2008; Shanok et al., 2019). Notably, the moderating effect of agent familiarity on emotion contagion was observed not just in autistic children but also in non-autistic children (Hudry & Slaughter, 2009). Nonetheless, autistic children might still be more sensitive to unfamiliar agents and unfamiliar situations.

Furthermore, it is worth mentioning that the parent-evaluated emotion contagion was only about how autistic children reacted to others’ negative emotions, whereas experimenter-evaluated emotion contagion was built upon autistic children’s aggregated performances of empathizing with positive and negative emotions (see Supplementary Table 7 for additional information). Like most empathy research, our study focused more on children’s abilities to empathize with others’ negative emotions (e.g. only negative emotions were involved in the evaluation of emotion contagion by parents and in the evaluation of prosocial actions by parents and experimenters) and did not distinguish children’s abilities of empathizing with different valences of emotions. Evidence shows that empathy for positive and negative emotions may engage different brain regions and involve different abilities (Morelli et al., 2015). For example, it was reported that while the tendency to empathize with others’ negative emotions was related to more personal distress and higher negative emotionality, the tendency to empathize with others’ positive emotions is related to higher positive emotionality and greater willingness and readiness for prosocial actions (Andreychik & Migliaccio, 2015; Murphy et al., 2018). Furthermore, although as discussed before, downregulating one’s own emotional distress is essential for facilitating empathic reactions to someone who experiences negative emotions, upregulating one’s own emotional arousals is often needed when empathizing with others’ joy and excitement (Brett et al., 2022). Future research in empathy should avoid this biased focus and broaden our understanding of empathy for both positive and negative emotions in typical and atypical development.

While the overall levels of empathy skills differed between autistic and non-autistic children, similar to non-autistic children, autistic children showed improvements in emotion acknowledgment and prosocial actions over time. Besides, the increase of prosocial actions was greater in autistic children than in non-autistic children. It is unclear why prosocial actions toward the experimenters increased more sharply in autistic children. Nonetheless, this finding supports the assumption that autistic children were not unempathetic and they had the potential to learn and to improve.

Not all empathy abilities increased with age. As expected, emotion contagion remained stable in both groups. Whereas appropriate levels of emotion contagion are crucial for motivating prosocial and compassionate actions toward others, excessive personal distress can disrupt the empathy process and make the person absorbed in self-concern. That children’s emotion contagion did not increase with age may reflect their enhanced ability to distinguish between self-distress and the distress of others (Hoffman, 2000). It may also have to do with their improved ability to regulate emotional arousals (Tousignant et al., 2017). Contrary to our expectation that children’s attention to others would increase with age, we found no age effect for parent-reported attention to others. Non-autistic children’s attention in the observational tasks actually dropped from Time 1 to Time 4. Possibly, with age, both autistic and non-autistic children became more proficient in evaluating others’ emotions, and thus they did not need to spend more time looking at others’ emotional displays. Their attention may even decrease if the situation becomes easier to process. This might be the case for non-autistic children in the observational tasks.

This study has the advantages of examining an autistic sample at a young age and using a multicomponent and multimethod approach to investigate the early development of empathy. Nonetheless, some caveats should be noted. First, emotion acknowledgment was measured only by a parent questionnaire, which evaluated the extent to which children understood their parents’ experiences of basic emotions. In daily life, social interactions involve multiple and complex emotional exchanges with not only parents but also peers and other adults. To capture the full picture of children’s development of emotion acknowledgment, future research should use multiple informants and examine not only basic emotion understanding, but also the understanding of complex emotions and mental states. Second, this study used the 19-item Empathy Questionnaire validated in a sample of Dutch children aged 1–5 years (Rieffe et al., 2010). However, more recent studies which have validated the Empathy Questionnaire in Italian, Spanish, and Japanese children suggest that a shorter 13-item version of the Empathy Questionnaire may be superior to the 19-item version in factor reliability and interpretability (Grazzani et al., 2017; Lucas-Molina et al., 2018; Takamatsu et al., 2021). To check whether the 13-item Empathy Questionnaire would yield different outcomes, we have rerun the analyses using the short version and found that the outcomes did not differ (see Supplementary Tables 5 and 6). Although this study chose the 19-item version because the age range and cultural background of the validation sample matched those of our sample, future research may consider using the 13-item version as it is less time-consuming, and the content may be more in line with the situation of young children nowadays. Third, emotion contagion was measured only by the subjective evaluations of parents and experimenters. To increase reliability, future research could add physiological measurements such as heart rates and skin conductance to the experimental paradigm. Fourth, this study only examined the effect of age, whereas other factors such as autistic traits, children’s cognitive abilities, and the social learning environment can also influence children’s empathy development. We have run post hoc analyses to explore the associations between children’s autistic traits and parent- and experimenter-evaluated empathy. The outcomes showed that children’s autistic traits were negatively associated with parent-reported empathy abilities such as emotion acknowledgment and prosocial actions (see Supplementary Table 4). Future research is warranted to advance our understanding of the protective and risk factors that contribute to the empathy development of autistic children. It should also be noted that the autistic sample included in this study did not have children with coexisting intellectual disabilities, had very few autistic girls, and the participants and their parents received regular supports from the intervention center where they were recruited for this study. The profile of other autistic groups may be different from what was depicted here.

Despite the limitations, the current study advanced our knowledge of empathy development in young autistic children. Compared with non-autistic children, autistic children showed lower levels of attention to others, emotion acknowledgment, and prosocial actions in empathy-provoking situations. However, these findings should be interpreted carefully. Autistic children’s empathy abilities could be underestimated due to a biased evaluation based on non-autistic standards, and the empathy problem may be bidirectional, where non-autistic people may encounter difficulty understanding and empathizing with autistic people. Future research should take into account the unique styles of empathy expression and reaction in autistic people, without necessarily setting the non-autistic styles as the norm. Importantly, we found that autistic children did show learning curves, which affirmed their potential for developing empathic abilities. Creating an inclusive social environment where autistic children feel welcomed and respected is likely to foster social learning opportunities equal to those encountered by non-autistic children. We suggest that inclusion and respect in an environment free of stereotyping, in turn, will encourage the development of empathic abilities among autistic children.

## Supplemental Material

sj-docx-1-aut-10.1177_13623613221117955 – Supplemental material for Toward feeling, understanding, and caring: The development of empathy in young autistic children Click here for additional data file. Supplemental material, sj-docx-1-aut-10.1177_13623613221117955 for Toward feeling, understanding, and caring: The development of empathy in young autistic children by Boya Li, Els Blijd-Hoogewys, Lex Stockmann, Ilaria Vergari and Carolien Rieffe in Autism
