# Supplementary material for: Toward feeling, understanding, and caring: The development of empathy in young autistic children
Source: Autism. 2022 Aug 23;27(5):1204–18. doi: 10.1177/13623613221117955 (PMC10291379; doi:10.1177/13623613221117955)
Supplement: sj-docx-1-aut-10.1177_13623613221117955 – Supplemental material for Toward feeling, understanding, and caring: The development of empathy in young autistic children [file sj-docx-1-aut-10.1177_13623613221117955.docx]

**Supplementary files**

Supplementary Table 1. Mean scores, standard deviations (SD) and reliabilities of parent-reported empathy of the autistic and non-autistic group at four time points.

|  | ***Autistic*** | | | | ***Non-autistic*** | | | |  |
| --- | --- | --- | --- | --- | --- | --- | --- | --- | --- |
|  | **Mean** | **SD** | **ω_t_** | **N** | **Mean** | **SD** | **ω_t_** | **N** |  |
| **Emotion contagion (0-2)** | |  |  |  |  |  |  |  |  |
| Time 1 | 0.32 | 0.34 | 0.85 | 54 | 0.30 | 0.32 | 0.89 | 118 |  |
| Time 2 | 0.34 | 0.38 | 0.86 | 50 | 0.30 | 0.31 | 0.88 | 49 |  |
| Time 3 | 0.38 | 0.42 | 0.91 | 45 | 0.26 | 0.29 | 0.80 | 41 |  |
| Time 4 | 0.38 | 0.36 | 0.83 | 31 | 0.22 | 0.30 | 0.91 | 33 |  |
| **Attention to others (0-2)** | | | | | | | | |  |
| Time 1 | 0.93 | 0.49 | 0.88 | 54 | 1.38 | 0.35 | 0.82 | 118 |  |
| Time 2 | 0.97 | 0.49 | 0.89 | 50 | 1.40 | 0.36 | 0.81 | 49 |  |
| Time 3 | 0.96 | 0.47 | 0.87 | 45 | 1.41 | 0.31 | 0.73 | 41 |  |
| Time 4 | 1.02 | 0.48 | 0.87 | 31 | 1.36 | 0.42 | 0.88 | 33 |  |
| **Prosocial actions (0-2)** | | | | | | | |  |  |
| Time 1 | 0.39 | 0.38 | 0.86 | 54 | 0.98 | 0.39 | 0.89 | 118 |  |
| Time 2 | 0.41 | 0.42 | 0.91 | 50 | 1.12 | 0.36 | 0.89 | 49 |  |
| Time 3 | 0.47 | 0.45 | 0.88 | 45 | 1.19 | 0.33 | 0.86 | 41 |  |
| Time 4 | 0.58 | 0.40 | 0.90 | 31 | 1.26 | 0.42 | 0.94 | 33 |  |
| **Emotion Acknowledgement (0-5)** | | | | | | | | | |
| Time 1 | 2.90 | 0.92 | 0.93 | 55 | 3.87 | 0.54 | 0.84 | 121 |  |
| Time 2 | 2.94 | 0.91 | 0.94 | 50 | 4.13 | 0.81 | 0.89 | 49 |  |
| Time 3 | 2.97 | 0.96 | 0.95 | 45 | 4.15 | 0.53 | 0.90 | 41 |  |
| Time 4 | 3.14 | 0.98 | 0.94 | 31 | 4.04 | 0.55 | 0.90 | 33 |  |

Note. The reliabilities were examined by McDonald’s ω_t_. Compared to Cronbach’s 𝛼, McDonald’s ω-statistics are more tolerant for assumption violations and have been proven the best reliability tests for both unidimensional and multidimensional measures (Revelle & Zinbarg, 2009).

Supplementary Table 2. Mean scores, standard deviations (SD) and reliabilities of observed empathy of autistic and non-autistic group at four time points.

|  | ***Autistic*** | | | | ***Non-autistic*** | | | |
| --- | --- | --- | --- | --- | --- | --- | --- | --- |
|  | **Mean** | **SD** | **ω_t_** | **N** | **Mean** | **SD** | **ω_t_** | **N** |
| **Emotion contagion (0-2)** | | | | | | | | |
| Time 1 | 0.64 | 0.48 | 0.90 | 61 | 0.83 | 0.47 | 0.84 | 145 |
| Time 2 | 0,63 | 0.53 | 0.83 | 50 | 0.89 | 0.53 | 0.80 | 51 |
| Time 3 | 0.68 | 0.39 | 0.78 | 47 | 0.73 | 0.44 | 0.70 | 48 |
| Time 4 | 0.59 | 0.46 | 0.83 | 43 | 1.13 | 0.43 | 0.74 | 44 |
| **Attention to others (0-2)** | | | | | | | | |
| Time 1 | 0.99 | 0.62 | 0.93 | 61 | 1.50 | 0.48 | 0.88 | 145 |
| Time 2 | 1.36 | 0.57 | 0.89 | 50 | 1.83 | 0.28 | 0.75 | 51 |
| Time 3 | 1.02 | 0.29 | 0.78 | 47 | 1.25 | 0.25 | 0.72 | 48 |
| Time 4 | 0.95 | 0.38 | 0.85 | 43 | 1.21 | 0.24 | 0.80 | 44 |
| **Prosocial actions (0-2)** | | | | | | | | |
| Time 1 | 0.22 | 0.28 | 0.87 | 60 | 0.31 | 0.35 | 0.82 | 144 |
| Time 2 | 0.40 | 0.40 | 0.71 | 50 | 0.54 | 0.41 | 0.84 | 51 |
| Time 3 | 0.64 | 0.36 | 0.66 | 47 | 0.41 | 0.33 | 0.55 | 48 |
| Time 4 | 0.49 | 0.29 | 0.64 | 42 | 0.57 | 0.40 | 0.64 | 43 |

Note. Reliabilities of the “Prosocial actions” scale at Time 3 (autistic: ω_t_=0.66; non-autistic: ω_t_=0.55) and at Time 4 (autistic: ω_t_=0.64; non-autistic: ω_t_=0.64) were low. A data inspection showed that the low reliabilities were due to the little variation of the ratings of some items at Time 3 and Time 4. A decrease of reliabilities is often observed in longitudinal studies. Sample attrition can make the remaining sample more similar, and the narrowing differences of the sample can cause a decrease of the measurement reliability (Bernardi, 1998).

Supplementary Table 3. Model fit indices and comparisons of the age models for empathy.

| **Parent reports** | | | | |
| --- | --- | --- | --- | --- |
|  | **Emotion contagion** | | | |
|  | AIC | BIC | -2LL | Χ^2^ statistics |
| ***Null model*** | 165.69 | 177.81 | 159.69 | - |
| ***Best age model*:** age | 155.43 | 171.37 | 147.43 | Χ^2^ (1) = 9.26, *p<*.001 |
|  | **Attention to others** | | | |
|  | AIC | BIC | -2LL | Χ^2^ statistics |
| ***Null model*** | 331.38 | 343.51 | 325.38 | - |
| ***Best age model:*** age, group | 292.52 | 312.44 | 282.52 | Χ^2^ (2) = 51.21, *p<*.001 |
|  | **Prosocial actions** | | | |
|  | AIC | BIC | -2LL | Χ^2^ statistics |
| ***Null model*** | 386.91 | 399.03 | 380.91 | - |
| ***Best age model:*** age, group | 264.66 | 284.58 | 254.66 | Χ^2^ (2) = 126.25, *p<*.001 |
|  | **Emotion acknowledgement** | | | |
|  | AIC | BIC | -2LL | X^2^ statistics |
| ***Null model*** | 817.56 | 829.72 | 811.56 | - |
| ***Best age model:*** age, group, | 706.84 | 726.81 | 696.84 | Χ^2^ (2) = 114.72, *p<*.001 |
| **Experimenter observation** | | | | |
|  | **Emotion contagion** | | | |
|  | AIC | BIC | -2LL | X^2^ statistics |
| ***Null model*** | 662.43 | 675 | 656.43 | - |
| ***Best age model:*** age, group | 636.06 | 656.91 | 626.06 | Χ^2^ (2) = 30.37, *p<*.001 |
|  | **Attention to others** | | | |
|  | AIC | BIC | -2LL | X^2^ statistics |
| ***Null model*** | 705.34 | 717.92 | 699.34 | - |
| ***Best age model:*** age, group,  age x group | 616.45 | 641.48 | 604.45 | Χ^2^ (3) = 41.43, *p<*.001 |
|  | **Prosocial actions** | | | |
|  | AIC | BIC | -2LL | X^2^ statistics |
| ***Null model*** | 423.79 | 436.34 | 417.79 | - |
| ***Best age model:*** age, group,  age x group | 379.49 | 404.47 | 367.49 | Χ^2^ (3) = 50.30, *p<*.001 |

Note. Models removed during the formal model-fitting procedures were not presented here. The χ^2^ statistics present the comparisons of the -2LL values between the best fitting models and the null models.

Supplementary Table 4. Correlations between SRS and parent- and experimenter-rated empathy in autistic children at time 1, 3, and 4. *R*-values are reported on the left and *p*-values on the right side of the slash; significant correlations are marked in bold.

| Parent reports | | | | | |
| --- | --- | --- | --- | --- | --- |
|  |  | *Emotion contagion* | *Attention to others* | *Prosocial actions* | *Emotion acknowledgement* |
| *SRS* | Time 1 | .103/.533 | -.097/.556 | **-.418**/.008 | **-.588**/.000 |
|  | Time 3 | .064/.674 | **-.329**/.037 | **-.558**/.000 | **-.720**/.000 |
|  | Time 4 | .272/.138 | -.197/.288 | **-.681**/.000 | **-.733**/000 |
| Experimenter observations | | | | | |
|  |  | *Emotion contagion* | *Attention to others* | *Prosocial actions* |  |
| *SRS* | Time 1 | .101/.537 | .052/.752 | .120/.460 |  |
|  | Time 3 | -.175/.245 | -.098/.516 | -.061/.689 |  |
|  | Time 4 | -.019/.928 | .061/.766 | .**417**/.038 |  |

Supplementary Table 5. Fixed and random effects of the best age models for parent-reported empathy (13-item EmQue).

|  | **Emotion contagion** | | | | **Attention to others** | | | |
| --- | --- | --- | --- | --- | --- | --- | --- | --- |
| **Fixed effects** | **Estimates** | **SE** | **CI [low, high]** | ***p*-value** | **Estimates** | **SE** | **CI [low, high]** | ***p*-value** |
| *Intercept* | .32 | .07 | [.19, .45] | <.001 | 1.49 | .07 | [1.35, 1.63] | <.001 |
| *age* | -.002 | .001 | [-.004, .0004] | .117 | .001 | .001 | [-.001, .003] | .297 |
| *group* | - | - | - | - | -.51 | .10 | [-.71, -.30] | <.001 |
| **Random effects** | **Estimates** | **SE** | **CI [low, high]** | **Wald’s Z** | **Estimates** | **SE** | **CI [low, high]** | **Wald’s Z** |
| *Residual* | .06 | .006 | [.19, .45] | 10.08 | .07 | .007 | [.06, .08] | 10.07 |
| *Intercept* | .44 | .05 | [.34, .55] | 8.29 | .37 | .05 | [.29, .47] | 8.04 |
|  | **Prosocial actions** | | | |  | | | |
| **Fixed effects** | **Estimates** | **SE** | **CI [low, high]** | ***p*-value** |  |  |  |  |
| *Intercept* | .96 | .08 | [.80, 1.11] | <.001 |  |  |  |  |
| *age* | .003 | .001 | [.0006, .005] | .013 |  |  |  |  |
| *group* | -.66 | .11 | [-.88, -.45] | <.001 |  |  |  |  |
| **Random effects** | **Estimates** | **SE** | **CI [low, high]** | **Wald’s Z** |  |  |  |  |
| *Residual* | .08 | .008 | [.07, .10] | 9.98 |  |  |  |  |
| *Intercept* | .42 | .05 | [.33, .54] | 7.86 |  |  |  |  |

Supplementary Table 6. Model fit indices and comparisons of the age models for parent-reported empathy (13-item EmQue).

|  | **Emotion contagion** | | | |
| --- | --- | --- | --- | --- |
|  | AIC | BIC | -2LL | Χ^2^ statistics |
| ***Null model*** | 502.83 | 514.96 | 496.83 | - |
| ***Best age model*:** age | 494.77 | 510.61 | 486.66 | Χ^2^ (1) = 10.17,  *p <* .002 |
|  | **Attention to others** | | | |
|  | AIC | BIC | -2LL | Χ^2^ statistics |
| ***Null model*** | 531.41 | 543.55 | 525.41 | - |
| ***Best age model:*** age, group | 503.68 | 523.61 | 493.68 | Χ^2^ (2) = 31.73,  *p <* .001 |
|  | **Prosocial actions** | | | |
|  | AIC | BIC | -2LL | Χ^2^ statistics |
| ***Null model*** | 628.82 | 640.95 | 622.82 | - |
| ***Best age model:*** age, group | 576.91 | 596.84 | 566.91 | Χ^2^ (2) = 55.91,  *p <* .001 |

Supplementary Table 7. Fixed and random effects of the best age models for experimenter-evaluated emotion contagion for positive and negative emotions.

| **Observed emotion contagion** | **Positive emotions** | | | | **Negative emotions** | | | |
| --- | --- | --- | --- | --- | --- | --- | --- | --- |
| **Fixed effects** | **Estimates** | **SE** | **CI [low, high]** | ***p*-value** | **Estimates** | **SE** | **CI [low, high]** | ***p*-value** |
| *Intercept* | .83 | .10 | [.64, 1.03] | <.001 | .79 | .06 | [.67, .90] | <.001 |
| *age* | .007 | .002 | [.003, .01] | .001 | -.001 | .001 | [-.004, .001] | .309 |
| *group* | -.20 | .10 | [-.39, -.004] | .045 | -.25 | .05 | [-.35, -.15] | <.001 |
| **Random effects** | **Estimates** | **SE** | **CI [low, high]** | **Wald’s Z** | **Estimates** | **SE** | **CI [low, high]** | **Wald’s Z** |
| *Residual* | .46 | .04 | [.39, .54] | 11.75 | .21 | .02 | [.18, .25] | 12.41 |
| *Intercept* | .19 | .04 | [.12, .30] | 4.34 | .02 | .01 | [.01, .07] | 1.77 |

Notes: Post-hoc analyses were run to check whether the results of experimenter-evaluated emotion contagion for positive emotions and for negative emotions would differ. The results did not differ much from the outcomes of aggregated emotion contagion for positive and negative emotions. In both conditions, autistic children were rated by experimenters as showing less emotion contagion than their non-autistic peers. Besides, the two groups did not differ in the developmental trajectories, and emotion contagion for negative emotions remained stable over time. However, an increasing trend was found in emotion contagion for positive emotions, namely, all children showed more emotion contagion for positive emotions as they grew older. This finding is consistent with the assumption that while downregulation of emotional arousals is needed when empathizing with others’ negative emotions, upregulation of emotional arousals may be needed when empathizing with others’ positive emotions (Brett et al., 2022). Notably, autistic children did not differ from non-autistic children in this trend of development.

**References**

Bernardi R. A. (1998). Sample attrition and Cronbach Alpha: A five-year longitudinal study. *Psychological Reports*, *82*(3_suppl), 1223-1231. <https://doi.org/10.2466/pr0.1998.82.3c.1223>

Brett J. D., Becerra R., Maybery M. T., Preece D. A. (2022). The Psychometric Assessment of Empathy: Development and Validation of the Perth Empathy Scale. *Assessment*. <https://doi.org/10.1177/10731911221086987>

Revelle, W., & Zinbarg, R. E. (2009). Coefficients alpha, beta, omega, and the glb: Comments on Sijtsma. *Psychometrika*, *74(*1), 145. <https://doi.org/10.1007/s11336-008-9102-z>
